# Deep Reinforcement Learning for Dynamic Obstacle Avoidance of Mobile Robots in Indoor Environments: A Review

**DOI:** 10.3390/s26154797

**Published:** 2026-07-28

**Authors:** Jiandong Zhao, Honghua Zhao, Benwang Li, Xuyin Gong

**Affiliations:** 1 School of Mechanical Engineering, University of Jinan, Jinan 250022, China; 202421100359@stu.ujn.edu.cn; 2 China Automotive Engineering Research Institute (Chongqing) Robot Testing Technology Co., Ltd., Chongqing 401120, China; gongxuyin@caeri.com.cn

**Keywords:** deep reinforcement learning, mobile robot, complex indoor environment, dynamic obstacle avoidance

## Abstract

**Highlights:**

**What are the main findings?**

- Systematically classifies DRL algorithms for indoor mobile robot dynamic obstacle avoidance.
- Analyzes core challenges and improved strategies across five key technical dimensions.

**What are the implications of the main findings?**

- Provides a unified reference framework for robot navigation and DRL algorithm selection.
- Clarifies promising future directions for indoor dynamic obstacle avoidance research.

**Abstract:**

The ability of mobile robots to avoid obstacles dynamically in indoor environments is a necessary condition for achieving autonomous planning and navigation. When dealing with unstructured and randomly dynamic indoor scenes, traditional obstacle avoidance algorithms have poor adaptability and low flexibility, making it difficult to handle environmental uncertainties. Deep Reinforcement Learning (DRL), with its efficient end-to-end decision-making, autonomous interactive learning capabilities, and proficiency in modeling complex dynamic systems, has emerged as a focal point of research in dynamic obstacle avoidance. This paper first presents the theoretical foundation of DRL, then categorizes the fundamental DRL algorithms for indoor dynamic obstacle avoidance into three main types: Value function-based, Policy-based, and Actor-Critic-based algorithms, while also introducing relevant algorithms. Furthermore, it addresses the core challenges encountered in indoor dynamic obstacle avoidance and summarizes various improvement strategies for the different basic algorithms, detailing their starting points and performance impacts. Finally, the paper outlines the development trends and future research directions in this domain. This review aims to serve as a systematic reference for the design and engineering application of DRL algorithms in dynamic obstacle avoidance for indoor mobile robots.

## 1. Introduction

Indoor mobile robots, as the core carriers of intelligent manufacturing and smart service scenarios, such as warehouse automated guided vehicles (AGVs), commercial service robots, robots for park/factory area inspection, and robots for hospital drug delivery, have been widely applied in various fields, including logistics and warehousing, commercial services, industrial inspection, and medical care [1]. The main operational requirement for this type of robot is to safely and efficiently achieve collisionless movement from the starting point to the target point in an unknown or somewhat unknown indoor environment. The dynamic obstacle avoidance capability, that is, the ability to avoid non-static threats such as randomly appearing pedestrians, mobile devices traveling in the same direction or opposite directions, temporarily piled obstacles, and movable facilities in real time [2,3]. Dynamic obstacle avoidance capability directly determines the level of autonomous operation of robots, their adaptability to the environment, and the practicality of implementation. It is a core indicator for measuring the intelligence level of indoor mobile robots [4].

The inherent attributes of indoor working scenarios bring many challenges to dynamic obstacle avoidance work: indoor spaces often have restricted working conditions, such as narrow passages, dense shelves, and blind spots at corners, which limit the maneuverable space for robots to avoid obstacles. The irregular passage of pedestrians, the interlaced operation of multiple mobile robots, and the sudden appearance of temporary obstacles cause the environmental state to have a high degree of randomness and dynamic time-varying [5]. Traditional indoor local obstacle avoidance algorithms have been widely deployed. Classic reactive methods, such as the Artificial Potential Field (APF) [6] and the Dynamic Window Approach (DWA) [7], enhance local planning efficiency owing to their real-time computational capabilities. Model-based frameworks like Model Predictive Control (MPC) [8] and the Timed Elastic Band (TEB) algorithm [9] are frequently employed to satisfy kinematic constraints and achieve trajectory optimization. Furthermore, geometric approaches like the Velocity Obstacle (VO) [10] method compute collision-free velocity spaces based on relative motion vectors. However, these traditional methods exhibit inherent limitations in highly randomized and unstructured indoor environments. APF frequently encounters local minima and severe trajectory oscillations within narrow passages. DWA and VO typically lack long-term predictive capabilities and rely heavily on the assumption of constant obstacle motion, which often fails against randomly behaving and interactive pedestrian trajectories. Concurrently, solving non-linear optimization problems under dense, time-varying constraints causes MPC and TEB to incur high real-time computational overhead, and their performance degrades significantly under unmodeled kinematic disturbances or inaccurate environmental priors. Therefore, relying solely on model-based analytical methods makes it difficult to satisfy the flexibility and multi-objective collaborative optimization required for modern indoor robotic operations [11]. This operational bottleneck has driven the paradigm shift toward data-driven approaches.

DRL is an intelligent decision-making technology that integrates the advantages of deep learning and reinforcement learning. It offers a brand-new technical approach to addressing the industry pain point of dynamic obstacle avoidance for indoor mobile robots and has become a current research hotspot and mainstream technical path in this field [12]. DRL takes the Markov Decision Process (MDP) [13] as the core modeling framework and transforms the work of dynamic obstacle avoidance for robots into a sequential decision optimization problem. By leveraging the powerful nonlinear feature extraction capabilities of neural networks, environmental features can be directly extracted from the raw data of airborne sensors, mainly composed of lidar and visual sensors, thereby completing the perception modeling related to complex indoor dynamic environments. Relying on the trial-and-error interaction and decision optimization mechanism of reinforcement learning, through the continuous interaction between the robot and the indoor environment, the core goal of “safe obstacle avoidance and efficient arrival” is achieved [14]. By adopting strategies of interactive exploration and autonomous learning, the end-to-end obstacle avoidance decision-making from the original perception input to the output of motion control instructions is ultimately achieved, breaking away from the reliance on environmental prior modeling and manual rules [15].

DRL still faces many challenges when applied to obstacle avoidance tasks. For instance, the inherent sparse reward feature of obstacle avoidance tasks makes it difficult for the model to converge [16]. From the perspective of generalization ability, the model’s ability to adapt to unknown dynamic environments and cross-scene migrations is relatively poor, making it difficult to handle complex situations in the real world, such as the arbitrary movement of obstacles and changes in environmental layout [17]. In terms of engineering implementation, the Partially Observable Markov Decision Process (POMDP) [18] in real obstacle avoidance scenarios is inconsistent with the basic assumption of complete observability of the algorithm and is prone to decision-making failure due to the limitation of the sensor’s field of view and the obstruction of obstacles [19].

In recent years, the integration of artificial intelligence has driven a proliferation of comprehensive reviews in the field of mobile robot navigation and intelligent control. Table 1 summarizes the primary analytical focus of recent representative surveys in this domain.

This paper exclusively targets the distinct challenge of dynamic obstacle avoidance in highly randomized indoor environments, systematically sorts out the research progress of DRL in the field of dynamic obstacle avoidance for indoor mobile robots, and clarifies the relevant theoretical basis and core algorithm system in this field [23], and classifies and summarizes the application adaptation of DRL in obstacle avoidance in different dynamic indoor scenarios. This paper analyzes the core challenges of current research from four dimensions: value assessment optimization, spatio-temporal multimodal perception, hierarchical mixing and expert experience control, and interaction safety perception [25]. At the same time, it sorts out the research trends in the field and predicts the future development direction, providing systematic academic references for researchers in the field of dynamic obstacle avoidance of indoor mobile robots.

**Figure 1:**
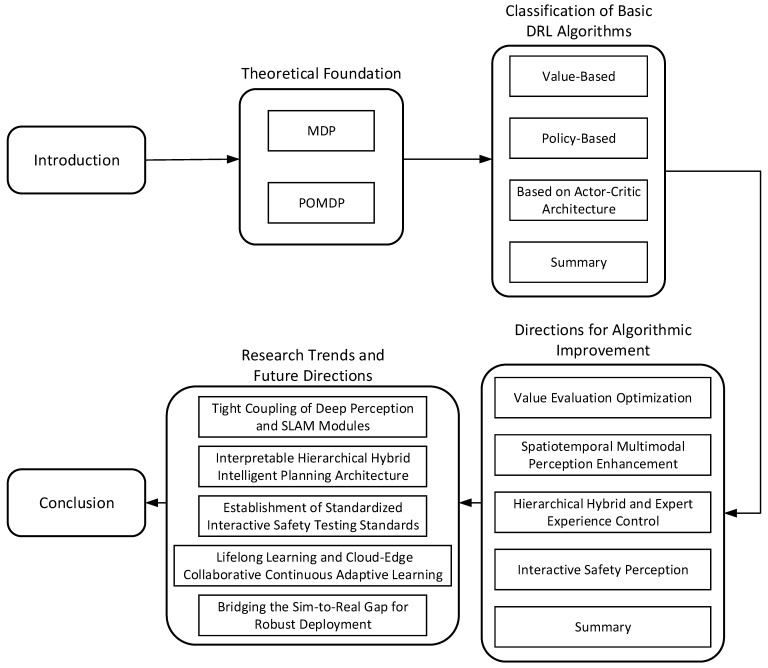
Article Structure diagram.

The structure of this article is shown in Figure 1.

## 2. The Theoretical Basis of Obstacle Avoidance in DRL Algorithms

All reinforcement learning algorithms are grounded in the mathematical framework of Markov Decision Processes (MDPs). This framework offers a standardized mathematical representation for the sequential decision-making of agents. Essentially, MDP is expressed in a five-tuple, reflecting the state characteristics and interaction forms of both agents when interacting with the environment. The various components of the MDP five-tuple are defined as follows [24]:

State: The state is defined as the direct representation of an agent’s position during its interaction with the environment. It $s_{t}$ quantifies the agent’s status at a given time t. Typically, the term S refers to the collection of states in which the agent exists, encompassing the range defined by the state. The states of a robot when avoiding obstacles include its own posture and position, speed parameters (line/angle), global environmental map, various (static/dynamic) obstacle positions and motion states, target point coordinates, and other elements [26].

Action: An action denotes the process by which an agent engages with its environment and makes decisions based on the information it has gathered and the current environmental context, ultimately achieving a specific goal. The actions taken by the agent in the t time step are denoted as $a_{t}$, and A usually represent the set of all actions available to the agent. There are two classification forms of the action space when mobile robots perform obstacle avoidance operations: the action space existing in a discrete manner and the action space existing in a continuous manner. The continuous action space can make the motion control effect smoother, and this method is generally adopted in dynamic obstacle avoidance scenarios [22].

Reward: This term denotes the value returned by the environment to the agent for the current step following a complete cycle of interaction. When setting rewards for dynamic obstacle avoidance tasks, the reward function is generally composed of three core modules: considerable positive rewards for successfully reaching the destination, huge negative losses from collisions, and gradient rewards considering the safety radius and heading effectiveness. Whether the reward function is scientifically set directly affects the efficiency and performance of obstacle avoidance strategy learning [27].

Discount rate: From the initial t time step, all recorded rewards are assigned a varying discount rate γ according to their temporal distance from this starting point, where 0≤γ≤1. As γ closer to 1 indicates that the agent prioritizes the evaluation of long-term benefits related to navigation and obstacle avoidance, thereby minimizing short-sighted decisions. Conversely, a rate γ closer to 0 signifies that the agent emphasizes immediate rewards.

Transition probability: This term refers to the probability distribution governing an agent’s transition from its current state to the subsequent state, typically denoted by a specific symbol P. In dynamic obstacle avoidance scenarios, state transitions involve the evolution of the robot’s own kinematic model and the occasional motion changes in dynamic obstacles, which implicitly exist when making obstacle avoidance decisions in dynamic environments [28].

During the interaction between an agent and its environment, the behavioral pattern of choosing a specific action in a particular state is called the agent’s policy. The strategy determines that the agent should take corresponding actions based on the current environmental state. There are two types of strategies: Deterministic Policy and Stochastic Policy. The expected return of a certain state within an interaction process is called the value of this state. The value function is the sum of the functions composed of the values of all states [13].

In the reinforcement learning training process, the agent’s task is to identify the optimal combination of strategies from a set of random strategies to maximize long-term rewards and achieve the highest value. The ideal solution may manifest as a single optimal strategy, although multiple strategies may exist. Each of these strategies can be articulated as follows $\pi^{*}(s)$. When the agent employs the optimal strategy for decision-making, the resulting state value function is the optimal state value function. As the agent transitions from one optimal strategy to another, the value associated with its action is referred to as the optimal action value function [29]. The mathematical relationship between the optimal state value function and the action value function is delineated by the Bellman optimality equation, expressed as:

(1) $$\left\{\begin{array}{l} V^{*}(s)=\underset{a\in A}{\max}\{R(s,a)+\gamma \sum_{s,s^{\prime }\in S}p(s^{\prime }|s,a)V^{*}(s^{\prime })\}\\ Q^{*}(s,a)=R(s,a)+\gamma \sum_{s,s^{\prime }\in S}p(s^{\prime }|s,a)\underset{a\in A}{\max}Q^{*}(s^{\prime },a^{\prime }) \end{array}\right.$$

The Bellman optimal equation is the core theoretical basis for reinforcement learning to find the optimal strategy and provides a mathematical basis for the iterative optimization of algorithms [29]. By using this equation, reinforcement learning algorithms can reverse-derive the optimal value of all predecessor states from the terminal target state and then determine the optimal action corresponding to each state. Ultimately, they can obtain an obstacle avoidance strategy that maximizes long-term cumulative rewards. This is the core mathematical support for various DRL algorithms in the field of indoor dynamic obstacle avoidance to achieve strategy optimization [30].

Considering the characteristics of indoor dynamic obstacle avoidance in real scenarios, the task must be modeled as a POMDP [18]. Due to factors such as limited sensor perception range, dynamic obstacle occlusion, an unknown environment, and measurement noise, the robot cannot acquire a complete global state $s_{t}$ of the environment and can rely only on local observations $o_{t}$ obtained through its sensors. Consequently, two core elements are introduced based on the MDP:

Observation space Ω: The set of all observations that the robot can acquire using sensors, $o_{t}\in \Omega$, which generally includes local information such as lidar ranging data, visual image data, the robot’s relative position/heading angle with the target point, and its own velocity.

Observation probability function O: It represents the probability distribution of the robot’s observations after the environment state transitions to the next state.

POMDP is a real mathematical model of indoor dynamic obstacle avoidance tasks and also the theoretical root of the core challenges in this field [31].

The differences between MDP and POMDP are shown in Table 2.

**Figure 2:**
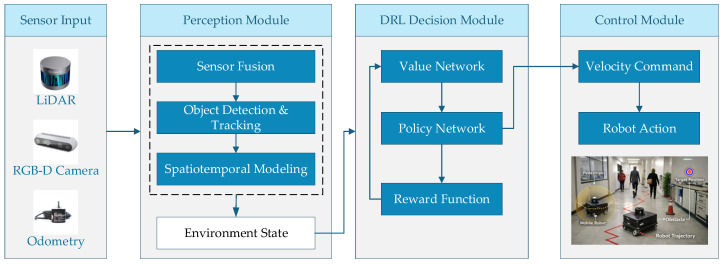
Overall technical framework diagram of indoor dynamic obstacle avoidance for robots based on DRL.

The overall technical framework for controlling robots to avoid obstacles in indoor dynamic environments using the DRL algorithm is shown in Figure 2.

## 3. The Classification and Scene Adaptability of the Basic Algorithm System of DRL

DRL, as an important branch in the field of artificial intelligence, has become a key technology for solving obstacle avoidance tasks in dynamic environments, relying on its learning ability in high-dimensional state spaces and its good adaptability to complex decision-making problems [14]. According to the differences in the underlying logic of strategy optimization, DRL is mainly divided into three systems: Value-based methods, Policy-based methods, and the Actor-Critic architecture, which integrates the strengths of both.

The Value-based methods evaluate the expected cumulative returns of each action in a specific state, indirectly obtaining the optimal strategy [32]. The Policy-based methods directly parameterize the policy and also use the gradient ascent method to find the optimal solution in the policy space, which makes them naturally suitable for handling continuous action spaces [30]. The Actor-Critic architecture combines the advantages of policy gradient (Actor) and value function approximation (Critic): the Actor outputs continuous actions based on the existing state, while the Critic’s job is to evaluate the value of the action and thereby reduce the variance of the policy gradient [33].

### 3.1. Value-Based DRL Algorithm

Deep Q-Network (DQN) is a pioneering algorithm proposed by DeepMind [32]. This algorithm successfully combines reinforcement learning and deep Convolutional Neural Networks (CNNs) for the first time, enabling agents to directly learn control schemes from high-dimensional sensor data input, such as raw pixels. To address the oscillation and divergence issues that are prone to occur when nonlinear networks approach the Q value, DQN has made two core modifications. First, experience replay: The algorithm stores the transfer tuples $e_{t}=\left(s_{t},a_{t},r_{t},s_{t+1}\right)$ of each time step in the experience pool and randomly selects a small batch of data during training, eliminating the temporal correlation of the observation sequence and making the distribution of the training data smoother. Second, independent target network: DQN generates Time Difference (TD) errors through an independent target network $\hat{Q}$ with relatively slow parameter updates, reducing the high correlation between the target value and the current predicted value.

The core optimized loss function is defined as the mean square error between the predicted Q value and the target Q value:

(2) $$L_{i}(\theta_{i})=E_{\left(s,a,r,s^{\prime })\sim U(D\right)}\left[{\left(r+\gamma \underset{a^{\prime }}{\max}\hat{Q}(s^{\prime },a^{\prime };\theta_{i}^{-})-Q(s,a;\theta_{i})\right)}^{2}\right]$$

Among them, $\theta_{i}$ represents the current network parameter, $\theta_{i}^{-}$ represents the target network parameter, and U(D) indicates uniform sampling from the experience pool.

The output layer of DQN corresponds to a discrete action space. During indoor obstacle avoidance tasks, it is applicable to robots that use omnidirectional wheel chassis and have action spaces that can be discretized (such as only including “forward, left turn, right turn, and stop”) to perform simple tasks. It can also carry out path planning for mobile robots based on raster maps [34]. For robots constrained by non-omnidirectional/Ackermann steering, discrete actions can cause jagged oscillations in the driving trajectory, making it impossible to achieve smooth, continuous speed and steering control. Moreover, in complex dynamic environments, the problem of overestimating the Q value can easily lead the robot to make aggressive decisions and result in collisions [35].

### 3.2. Policy-Based DRL Algorithm

#### 3.2.1. TRPO Algorithm

Trust Region Policy Optimization (TRPO) algorithm [36] is a classic algorithm proposed to overcome the problems that the step size of traditional policy gradient methods is difficult to control, and the performance of policy update is poor. Within the scope of the standard policy gradient, considering that the objective function is highly nonlinear in the parameter space, a single large parameter update may cause a significant decline in the policy performance of the agent. To theoretically ensure that the policy can have a monotonically increasing performance improvement each time it is updated, TRPO introduces the constraint concept of “Trust Region”. TRPO does not directly limit the step size in the parameter space but calculates the Kullback–Leibler Divergence ($D_{KL}$) between the old and new policy distributions to strictly constrain the magnitude of the policy update range.

From a mathematical perspective, the core of TRPO is to optimize the strategy by maximizing a substitution advantage objective function. Meanwhile, subject to the hard constraint of KL divergence, the constrained optimization problem of this algorithm is defined as:

(3) $$\underset{\theta }{maximize}E_{s\sim \rho_{\theta_{old}},a\sim q}\left[\frac{\pi_{\theta }(a|s)}{q(a|s)}Q_{\theta_{old}}(s,a)\right]$$

(4) $$subject\;to\;E_{s\sim \rho_{\theta_{old}}}\left[D_{KL}\left(\pi_{\theta_{old}}(\cdot |s)∥\pi_{\theta }(\cdot |s)\right)\right]\leq \delta$$

When indoor mobile robots carry out dynamic obstacle avoidance tasks, the advantages of TRPO over other algorithms are: It ensures monotonous improvement of theory-level strategies and high training stability. When robots perform high-risk obstacle avoidance exploration in complex indoor environments (such as crowded narrow passages), this stability can effectively prevent catastrophic collisions and behavioral degradation caused by a single update of network parameters. However, TRPO has very obvious limitations: when solving second-order constrained optimization problems, the computational complexity is very high, the computational cost is huge, and the implementation process is complicated. For robots that are only equipped with lightweight edge computing devices and have limited on-board computing resources, TRPO finds it difficult to meet the requirements of real-time decision-making and efficient computing in actual engineering [37].

#### 3.2.2. PPO Algorithm

The Proximal Policy Optimization (PPO) algorithm [30] was proposed as an efficient policy gradient method to address the computational limitations of the TRPO algorithm. Although TRPO theoretically guarantees monotonic policy improvement, it relies on conjugate gradient methods to solve a second-order constrained optimization problem, resulting in complex implementation and high computational costs. The PPO algorithm aims to attain the data efficiency and reliable performance of TRPO while using only straightforward first-order optimization.

The core innovation of PPO lies in the introduction of a novel surrogate objective function with clipped probability ratios. Let the probability ratio between the new and old policies be:

(5) $$r_{t}(\theta )=\frac{\pi_{\theta }(a_{t}|s_{t})}{\pi_{\theta_{old}}(a_{t}|s_{t})}$$

The core clipped surrogate objective of PPO is then defined as:

(6) $$L^{CLIP}(\theta )={\hat{E}}_{t}\left[\min(r_{t}(\theta ){\hat{A}}_{t},\mathrm{clip}(r_{t}(\theta ),1-\epsilon ,1+\epsilon ){\hat{A}}_{t})\right]$$

where ${\hat{A}}_{t}$ is the estimator of the advantage function, and ϵ is a hyperparameter used to constrain the magnitude of the policy update. In this objective function, the clip function modifies the surrogate objective by clipping the probability ratio, thereby removing the incentive for moving $r_{t}$ outside the interval $\left[1-\epsilon ,1+\epsilon \right]$. By taking the minimum of the unclipped and clipped terms, the final objective forms a pessimistic estimate of the unclipped objective regarding policy performance. This mechanism ignores the change in the probability ratio when it would improve the objective, but includes it when it makes the objective worse, effectively preventing destructively large policy updates.

When indoor mobile robots execute dynamic obstacle avoidance tasks, the PPO algorithm demonstrates significant application value. Compared to TRPO, PPO is much simpler to implement, more general, and empirically exhibits better sample complexity. It not only excels in continuous control tasks, enabling the output of smooth chassis velocity and steering commands, but also is compatible with architectures that require parameter sharing between the policy and value functions. Because PPO strikes a favorable balance among sample complexity, algorithmic simplicity, and training wall-time, it has emerged as one of the preferred mainstream baseline algorithms for handling complex dynamic obstacle avoidance and intelligent robot navigation [23].

### 3.3. DRL Algorithm Based on Actor-Critic Architecture

#### 3.3.1. DDPG Algorithm

Deep Deterministic Policy Gradient (DDPG) algorithm [15] is a model-free, Off-policy Actor-Critic algorithm that can meet the requirements of controlling robots to perform high-dimensional continuous actions. DDPG incorporates the successful experiences of DQN: experience replay and target network, into the Deterministic Policy Gradient (DPG) framework. To ensure the effectiveness of the algorithm’s exploration under the deterministic strategy, DDPG superimposes the time-dependent Ornstein-Uhlenbeck (OU) noise into the deterministic actions of the Actor network output. Additionally, its target network adopts the “Soft target updates” approach, namely $\theta^{\prime }\leftarrow \tau \theta +(1-\tau )\theta^{\prime }(\tau \ll 1)$, to ensure the smoothness of the target value update iteration.

The loss function of the Critic network is to minimize the mean square error of the Bellman residual:

(7) $$L(\theta^{Q})=E\left[{\left(r+\gamma Q^{\prime }\left(s^{\prime },\mu^{\prime }\left(s^{\prime }|\theta^{\mu^{\prime }}\right)|\theta^{Q^{\prime }}\right)-Q(s,a|\theta^{Q})\right)}^{2}\right]$$

The update of the Actor network is based on the gradient chain rule of the Critic:

(8) $$\nabla_{\theta^{\mu }}J\approx E\left[\nabla_{a}Q(s,a|\theta^{Q}){|}_{a=\mu (s)}\nabla_{\theta^{\mu }}\mu (s|\theta^{\mu })\right]$$

DDPG can directly output very precise chassis motor control instructions, making it highly suitable for wheeled mobile robots with kinematic constraints. However, the deterministic strategy of this algorithm is relatively slow to respond when facing highly dynamic indoor obstacles (such as sudden intrusions of pedestrians), and DDPG is highly sensitive to the settings of hyperparameters and noise. The Q value often underestimates seriously, causing the robot to frequently misjudge and result in collisions when facing complex intersections [38].

#### 3.3.2. A3C/A2C Algorithms

The Asynchronous Advantage Actor-Critic (A3C) algorithm [32] can run several agent environment replicas in parallel on multiple threads of the CPU, and the replicas are still independent of each other. This algorithm can also perform asynchronous updates of the entire network parameters by using the accumulated gradients. This asynchronous parallel mechanism is of great help in breaking the temporal correlation of observed data, eliminating the need for a large and time-consuming experience replay pool. While maintaining strategies and value estimates, A3C also uses n-step returns to update both. Advantage Actor-Critic (A2C) [39] is the synchronous version of A3C. It waits for all the parallel working nodes to complete the experience collection and then uniformly updates the gradient based on the collected experience data. The advantage of doing so is that it can make more efficient use of GPU resources. Both of the two algorithms mentioned above use the dominance function $A\left(s,a\right)=Q\left(s,a\right)-V\left(s\right)$ to guide the update of the policy gradient.

Combining the policy entropy $H(\pi (s_{t};\theta ))$ as the regularization term, the core objective function (the expected objective to be maximized) of the Actor network (with a parameter of θ) is:

(9) $$J(\theta )=\log\pi (a_{t}|s_{t};\theta )\left(R-V(s_{t};\theta_{\nu })\right)+\beta H\left(\pi (s_{t};\theta )\right)$$

The corresponding policy gradient update formula is:

(10) $$\nabla_{\theta }J(\theta )=\nabla_{\theta }\log\pi (a_{t}|s_{t};\theta )A(s_{t},a_{t})+\beta \nabla_{\theta }H\left(\pi (s_{t};\theta )\right)$$

A3C/A2C rely on parallel environmental replicas to collect data, which enables them to conduct indoor obstacle avoidance simulation training on physical simulation platforms. However, due to the inability to “open multiple replicas” in the real world, when they are actually deployed to physical robots, their drawback of low sample utilization will be exposed and magnified infinitely. Meanwhile, the asynchronous update mechanism is also prone to causing instability in policy execution on real airborne computing platforms [34].

#### 3.3.3. SAC Algorithm

Soft Actor-Critic (SAC) algorithm [40] is an Off-policy algorithm that adopts the Maximum Entropy reinforcement learning framework, which is different from traditional reinforcement learning that only pursues maximizing the expected cumulative return. The SAC algorithm drives the agent to maximize its policy distribution entropy (randomness) while ensuring high returns. This mechanism endows the algorithm with strong exploration capabilities and robustness against environmental noise. SAC uses a double-Q network architecture to alleviate positive bias and trains the value function and policy network by minimizing the soft Bellman residual. The core objective function of it is:

(11) $$J(\pi )=\sum_{t=0}^{T}E_{(s_{t},a_{t})\sim \rho_{\pi }}[r(s_{t},a_{t})+\alpha H\left(\pi (\cdot |s_{t})\right)]$$

SAC is currently one of the optimal algorithms for handling highly dynamic and crowded indoor environments. Due to the randomness of its strategy, when encountering dynamic obstacles and pedestrians that suddenly intrude into the driving route, the robot’s ability to avoid risks and recover, as well as its multi-path selection ability, far exceeds those of deterministic strategies. It can fully utilize various samples in complex indoor environments, successfully avoiding obstacles while improving the trajectory smoothness [41].

#### 3.3.4. TD3 Algorithm

Twin Delayed Deep Deterministic Policy Gradient (TD3) Algorithm [42] is an optimized and improved algorithm for the common problems of “overvaluation” and “error accumulation” in continuous control methods based on Actor-Critic such as DDPG. In the continuous action space, the temporal difference update has a bootloader characteristic. This characteristic continuously amplifies the tiny errors of the function approximator, leading the update of the policy network to become suboptimal or even divergent. To suppress these unstable situations from theoretical and architectural roots, the TD3 algorithm introduces three core mechanisms:

First, clipped double Q-learning. TD3 contains two Critic value networks (with parameters of $\theta_{1}$ and $\theta_{2}$ respectively) whose initialization processes are independent of each other. This design enables the algorithm to choose the smaller predicted value given by the two when calculating the TD target value. This pessimistic estimation approach directly breaks the feedback loop of overestimation. The formula for calculating the target value y is:

(12) $$y=r+\gamma \underset{i=1,2}{\min}Q_{\theta_{i}^{\prime }}(s^{\prime },\tilde{a})$$

Here, $Q_{\theta_{i}^{\prime }}$ represents the predicted value of the target value network. The updated objective of the Critic network is to minimize the mean square Bellman error:

(13) $$J(\theta_{i})=E_{(s,a,r,s^{\prime })\sim B}\left[{\left(y-Q_{\theta_{i}}(s,a)\right)}^{2}\right],i=1,2$$

Second, the processing of target policy smoothing regularization. The deterministic strategy is very prone to overfitting, resulting in a peak in the value function estimation [43]. To smooth out the local fluctuations of the value function, when TD3 calculates the target action $\tilde{a}$, it adds a truncated normal distribution noise ε to the output of the target Actor network $\pi_{\Phi^{\prime }}$, therefore:

(14) $$\tilde{a}=\pi_{\Phi^{\prime }}(s^{\prime })+\varepsilon ,\varepsilon \sim clip(N(0,\sigma ),-c,c)$$

This method adds a mandatory requirement for the algorithm to evaluate the value of an action: taking the value within the action neighborhood into account, thereby better suppressing local disturbances in the action space [44].

Third, delayed policy updates. To avoid updating the parameters of the Actor network when the value estimation of the Critic network is inaccurate, which would cause the strategy to fluctuate, the TD3 algorithm reduces the update frequency of the Actor network and all target networks. The Deterministic Policy gradient update formula for the Actor network is (guided only by the first Critic $Q_{\theta_{1}}$):

(15) $$\nabla_{\Phi }J(\Phi )=E_{s\sim B}[\nabla_{a}Q_{\theta_{1}}(s,a){|}_{a=\pi_{\Phi }(s)}\nabla_{\Phi }\pi_{\Phi }(s)]$$

When the indoor mobile robot is performing dynamic obstacle avoidance tasks, the pessimistic estimation mechanism of TD3 enables it to have the ability to avoid obstacles more conservatively and safely when encountering them. The target strategy smoothing mechanism achieves smooth and continuous steering control of the output instructions of the robot chassis. Due to its excellent trajectory tracking stability, TD3 is suitable for indoor fine navigation tasks with low tolerance requirements, such as narrow corridor passage and high-precision transportation of medical supplies [44]. Because the TD3 needs to maintain six deep neural networks simultaneously inside, it poses relatively high hardware requirements for the on-board GPU memory and inference computing power of the robot [45].

### 3.4. Classification of Basic Algorithm Systems and Summary of Scenario Adaptability

The classification of the basic algorithm system of DRL and the scene adaptability summary of the corresponding algorithms are shown in Table 3.

## 4. Improved DRL Algorithm for Indoor Dynamic Obstacle Avoidance

The performance of native DRL algorithms in an ideal static environment has been verified. However, when dealing with dynamic and disturbed indoor complex scenes, they generally have fatal vulnerabilities such as policy oscillation, blind spots in perception, and poor generalization efficiency. In recent years, research has mainly focused on four dimensions: value assessment optimization, spatio-temporal multimodal perception, hierarchical hybrid architecture, expert experience control, and interaction security perception. Many targeted improvement algorithms have been provided. Below, some representative improvement algorithms are selected as examples for illustration.

### 4.1. Value Assessment Optimization

When dealing with the local dynamic obstacle avoidance task of complex indoor maps, the reinforcement learning algorithm is prone to overestimation bias when calculating the target Q value, resulting in insufficient assessment of high-risk states by the robot. The Double DQN (DDQN) algorithm [46] adopts a dual architecture of selecting actions in the online network and evaluating values in the target network. It successfully disassembles action selection and evaluation, fundamentally reducing the overestimation of Q values. The Dueling DQN algorithm [47] decomposes the Q value into state value and action advantage, enabling the strategy to converge more quickly. Meanwhile, for important sparse samples such as “collisions” in indoor obstacle avoidance, this algorithm determines the priority of sample experience, accelerating the network’s learning of high-value samples and improving the efficiency of state evaluation under redundant actions.

To enhance the guiding role of the reward signal, the DM-DQN algorithm [48] innovatively introduces logarithmic strategy regularization to the immediate reward. Based on integrating the advantages of the above algorithms, the PER-D2MQN algorithm [49] introduces an experience replay mechanism, systematically solving the problems of slow convergence and low sample efficiency of the DQN system in complex dynamic obstacle avoidance. The RND3QN algorithm [50] combines the N-step reward mechanism with the Dueling Double DQN structure, effectively balancing the bias and variance of value estimation, and accelerating the credit allocation speed under delayed rewards. While suppressing the overestimation of Q values, this algorithm improves the learning effectiveness of state value and action advantage. The algorithm should be made more stable in convergence and more efficient in sample utilization.

In the field involving continuous control, to address the challenges of insufficient exploration and sparse rewards, the NM-TD3 algorithm [51] adopts a dynamic hybrid exploration strategy, combining Gaussian noise with OU noise, which enhances the exploration stability and training efficiency of the continuous action space during path planning. The ASAC algorithm [52] embeds the dominance function within the SAC Maximum Entropy framework, reducing the variance of the policy gradient through baseline correction. While maintaining the benefits of entropy regularization exploration, it enhances the convergence speed and sample utilization rate. The LP-TD3 algorithm [53] simultaneously embeds the Priority Experience Replay (PER) and the Intrinsic Curiosity Module (ICM) within the TD3 framework. The ICM integrates external rewards with internal rewards, stimulating the robot to actively explore unknown areas and overcoming the problem of learning stagnation caused by blind spots.

While the aforementioned value assessment optimizations effectively address the core flaws of baseline DRL, they introduce distinct engineering trade-offs across their subcategories. First, architecture decoupling (e.g., DDQN, Dueling DQN) increases the structural complexity of the network, demanding more meticulous hyperparameter tuning. Second, replay and reward optimizations (e.g., PER) severely consume memory bandwidth due to the frequent updating of prioritized sampling trees, creating an I/O bottleneck for real-time edge computing. Finally, introducing hybrid exploration and intrinsic curiosity modules, while preventing the robot from stagnating in unknown regions, often causes unstable, jittery exploratory actions during early training phases, requiring a delicate balance between sample efficiency and physical control stability.

### 4.2. Enhancement of Spatio-Temporal Multimodal Perception

Indoor dynamic obstacle avoidance is fundamentally a POMDP, and a single static frame cannot infer the movement trajectory of dynamic obstacles. To endow the algorithm with memory of time series, the GRU-DDQN algorithm [54] proposes to combine recurrent neural networks and DDQN, enabling robots to use historical time series for autonomous navigation and obstacle avoidance. The SAC-LSTM algorithm [55] embeds LSTM in the continuous control strategy to implicitly predict the trajectory of dynamic obstacles and handle more complex mapless navigation. The BMTD3 algorithm [56] employs Bidirectional Gated Recurrent Units (BiGRU) to obtain deep sequence memory and constructs a Multi-Head Attention mechanism in the network to extract different key historical features, effectively alleviating the problems caused by incomplete environmental observability. The speed at which the network responds to changes in complex dynamic scenarios and the accuracy of value estimation have been enhanced.

In the front-end fusion part of multi-sensor fusion, the CNN-DQN algorithm [57] directly extracts high-dimensional spatial features from the grid map based on sensor data. To eliminate the differences in cross-modal data features, the CMADRL algorithm [58] proposed a method for dynamic fusion of lidar point clouds and RGB-D depth images, which solved the blind spots in the “vision” of robots.

When in an environment with dense obstacles, the attention-enhanced PPO algorithm [59] employs CBAM and EMA multi-scale attention modules, enhancing the sensitivity to high-threat obstacles. The GAP_SAC algorithm [60], by means of a gating mechanism, enables the network to focus on key environmental features and, in combination with heuristic rewards, solves the problem of slow convergence speed of SAC in narrow dynamic channels. The CAM-RL algorithm [61] proposes to enhance the algorithm’s attention to the relative dependency of the crowd, thereby improving the avoidance success rate of the robot in a crowded crowd environment. What belongs to the more cutting-edge category is that the end-to-end Spatio-temporal Transformer DRL [62] proposes to perform parallel processing on multi-obstacle interaction relying on the global self-attention mechanism, achieving active pre-judgment obstacle avoidance.

Critical Reflection: The enhancement of spatio-temporal multimodal perception theoretically resolves the POMDP bottleneck but faces severe computational challenges during physical deployment. For temporal prediction modules (e.g., LSTM/BiGRU), retaining long-term historical sequences exponentially increases inference latency. For cross-modal feature fusion, aligning high-dimensional data from disparate sensors (e.g., LiDAR and RGB-D) at the hardware level introduces synchronization delays and high-power consumption. Furthermore, while attention-enhanced prediction mechanisms (e.g., Transformers) successfully extract key threats in dense crowds, executing massive attention matrices on lightweight onboard edge computers (like Jetson Nano) makes it exceedingly difficult to maintain the control frequency required for safe dynamic avoidance.

### 4.3. Layered Mixing and Expert Experience Control

To address the chassis control oscillation caused by pure end-to-end output, building a hybrid architecture system is a commonly used approach in current engineering. The IDDPG-IAPF algorithm [63] takes the Improved Artificial Potential Field (IAPF) method as expert prior experience to assist DDPG in learning obstacle avoidance strategies and formulates a hierarchical strategy switching mechanism to adapt to the highly dynamic warehousing and inspection environment. In view of the shortcomings of the traditional potential field method, such as being prone to falling into local optimum and having difficulty avoiding traps and obstacles, the VDPF-TD3 algorithm [64], that is, the DRL hybrid framework combined with the variable direction potential field, was proposed. This framework designs an obstacle classification algorithm. When encountering traps and obstacles, the DRL algorithm takes over control of the robot. Combining a variable-direction potential field enables the robot to quickly escape from the local optimal solution, taking into account the stability of traditional algorithms and the flexibility of reinforcement learning algorithms.

In dealing with global and local collaborative planning problems, the A*-DQN-DWA algorithm [65] utilizes an improved A* to ensure global reachability. Under the condition of jointly using the DWA algorithm with kinematic constraints, DQN dynamically identifies local obstacle avoidance sub-targets and simultaneously generates smooth control instructions. The SAC-PID algorithm [66] utilizes SAC to receive the relevant dynamic information of the mobile robot as input and simultaneously provides the optimal parameters of the incremental PID controller to achieve real-time compensation for the path tracking error of the mobile robot. The CNN-DQN-B algorithm [57] further post-processes discrete actions by introducing B-spline curves, improving the smoothness of obstacle avoidance.

When performing complex indoor inspection tasks, multi-task learning conflicts may occur due to the existence of two tasks, namely “dynamic obstacle avoidance” and “target tracking”. The SSRL-PPO algorithm [67] decomposes the complex tasks into independent sub-scenarios and achieves model transformation with a finite state machine, effectively solving the learning conflict problem. Meanwhile, the completion rate of the tasks has been greatly improved. Furthermore, flat DRL policies often struggle with complex, long-horizon indoor navigation tasks. Hierarchical Deep Reinforcement Learning (HDRL) addresses this by decoupling high-level cognitive exploration from low-level reactive obstacle avoidance. Recent studies on spatial memory-augmented visual navigation utilize HDRL to significantly improve generalization and success rates in unseen environments [68].

Hierarchical hybrid architectures present the most feasible path for near-term industrial applications, yet they harbor inherent systemic flaws across different fusion levels. First, relying on expert prior guidance exposes the “suboptimal trap,” inherently capping the DRL agent’s performance upper bound to the level of the traditional algorithm. Second, low-level hybrid control (e.g., switching between DRL and DWA/PID) requires tedious manual tuning of heuristic switching rules, partially compromising the “end-to-end” autonomy. Finally, while task decoupling (e.g., HDRL or SSRL) mitigates multi-task conflicts, the transition logic between high-level cognitive layers and low-level reactive layers is often brittle, easily causing behavioral freezing or decision delays when environmental states oscillate near the switching thresholds.

### 4.4. Interactive Security Perception

As robots enter the spaces where human activities take place, they need to meet the conditions set for human–machine interaction safety technology. Estrella Elvia Montero et al. [69] proposed a dynamic warning range. In this area, circular sectors are set around the robot according to the human step length and speed. The warning area is implemented when the robot is doing DRL training to ensure a safe distance between the robot and the human. To set a hard safety range at the bottom layer of the algorithm, Long et al. [70] integrated a human risk predictor based on Monte Carlo simulation into the decision-making unit to select the safest path from among the candidate paths. Sun et al. [71] proposed a novel risk-aware DRL approach. This method incorporates conditional risk value into the objective function, enabling the robot to actively prioritize avoiding pedestrians with a higher collision risk and reducing the collision probability between the robot and pedestrians during the unknown exploration process. To ensure absolute physical safety, the Differential Safe Reinforcement Learning (DSRL) algorithm [72] incorporates hard constraints directly into policy optimization, strictly bounding risky exploratory behaviors during both training and deployment.

To accelerate the convergence of the model in the new environment, Zhu et al. [73] proposed a self-supervised DRL method that directly learns control instructions from the original depth image data, ensuring the safety of collaborative work between robots and humans in unstructured and complex environments. Additionally, for human-populated spaces, Inverse Reinforcement Learning (IRL) enables robots to infer underlying reward structures directly from expert human demonstrations, facilitating socially compliant and human-like collision avoidance without complex manual reward design [74]. Mei et al. [75] proposed a zero-shot hierarchical reinforcement learning algorithm, which endows robots with the generalization ability to avoid unknown obstacles based on semantic associations in a brand-new environment.

For the multi-robot collaborative operation scenarios in complex spaces, Zhao et al. [76] designed a high-performance multi-robot collaborative exploration strategy that combines Voronoi diagrams with multi-objective cost function models. It also integrates an obstacle avoidance algorithm based on DRL, enabling multi-machine collaborative exploration work without collision in unknown environments. The MADDPG-LSTM Actor algorithm [77] takes the continuously observed data in time as the input of the policy network and simplifies the input of the evaluation network, solving the problem of the lack of timing in traditional MADDPG. It performs well in multi-machine formation control and obstacle avoidance. For complex human–machine mixed traffic scenarios with numerous pedestrians, the MARL algorithm [78] employs a social encoder based on spatio-temporal graph (TSG) correlation, enabling multiple agents to understand social relationships. Meanwhile, it introduces a K-step forward-looking reward mechanism within the framework of multi-robot reinforcement learning to avoid short-sighted and invasive motion trajectories.

Addressing interactive safety issues is a primary prerequisite for human–robot coexistence, yet current solutions face strict theoretical and practical limitations. First, imposing hard safety constraints (such as DSRL or warning zones) often triggers the issue of the robot halting indefinitely in dense crowds to strictly avoid violating conservative risk thresholds. Second, interactive response enhancement methods (such as Inverse Reinforcement Learning, IRL) bypass complex reward engineering but rely heavily on the quality and quantity of human expert demonstrations, exhibiting poor generalization capabilities. Finally, interactive collaboration compliance models (such as MARL and social encoders) struggle to cope with non-stationarity; as the number of interacting pedestrians or robots increases, achieving real-time collaborative convergence becomes increasingly difficult.

### 4.5. Challenges and Solutions: Summary of Indoor Dynamic Obstacle Avoidance

Table 4 summarizes the core challenges faced in realizing real-time obstacle avoidance for mobile robots using DRL methods, as well as the corresponding improved algorithms and their targeted solutions to address these core challenges. Specifically, the challenges are classified into four key technical dimensions consistent with the full text, and the core design ideas and performance effects of each improved algorithm are sorted out accordingly.

Table 5 summarizes the performance of representative algorithms with superior obstacle avoidance capabilities selected from each subclass. It is important to note that since these algorithms were evaluated in vastly different environments—ranging from Atari games and simple static/dynamic obstacle scenes to dense crowds and unseen complex environments—this table does not serve as a direct quantitative performance ranking. Instead, it is intended as a qualitative summary of algorithmic characteristics and application scenarios. Its primary objective is to provide methodological inspiration and reference for researchers when applying DRL to various specific obstacle avoidance tasks. To enhance readers’ understanding of the various algorithms depicted in the table, this article also includes a set of algorithm architecture diagrams in Appendix A.

## 5. Research Trends and Future Directions

Based on the analysis of existing challenges, future research on DRL for complex indoor dynamic obstacle avoidance will break through the single algorithm optimization level and move towards a new stage of system-level integration and standardized norms. The following summarizes four hot research directions in this field for the future.

### 5.1. Tight Coupling of Depth Perception and SLAM Module

The future obstacle avoidance system architecture needs to focus on exploring the deep integration of DRL and Simultaneous Localization and Mapping (SLAM) systems [79]. To achieve tight coupling, future research must move beyond mere data sharing and establish concrete algorithmic mechanisms. For instance, SLAM uncertainty can be directly integrated into the DRL reward function. Using the pose covariance or mapping uncertainty output by the SLAM backend as a penalty term can guide the DRL policy to avoid feature-poor regions or reduce speed when localization confidence drops [80]. Additionally, active perception for obstacle avoidance is a practical direction. DRL actions can concurrently control chassis velocity and sensor orientation. This allows the robot to actively track dynamic obstacles while keeping sufficient static landmarks in the field of view, thereby ensuring robust SLAM performance during avoidance maneuvers [81].

Moreover, the potential conflicts and vulnerabilities of this coupled system must be carefully analyzed. A primary challenge is the error chain among localization drift, map consistency, and policy reliability. In highly dynamic indoor scenes, moving pedestrians often occlude static features, causing SLAM localization drift. This drift affects the robot’s coordinate perception and corrupts map consistency, causing the DRL agent to act on false state representations, which in turn degrades policy reliability. Another issue is the inherent perceptual conflict: SLAM treats dynamic objects as geometric noise to be rejected, while DRL must track these specific objects to avoid collisions. Future architectures must employ flexible decoupling front-ends to serve both needs simultaneously. Finally, the impact of SLAM loop closures must be mitigated. During loop closure, the instant pose jump in the global coordinate system can cause destructive, discontinuous spikes in the DRL action output. Therefore, designing DRL policies that operate in local reference frames or employing pose-smoothing buffering mechanisms is critical for safe real-world deployment.

### 5.2. Explainable Hierarchical Hybrid Intelligent Planning Architecture

The end-to-end DRL model is prone to trajectory oscillations and unstable control. In the future, a hybrid architecture that integrates DRL with traditional obstacle avoidance algorithms should be further promoted to address these issues. Specifically, it is to provide kinematic constraints and training safety experience by using traditional obstacle avoidance algorithms such as APF and DWA. At the same time, it combines expert knowledge and human experience and is combined with the autonomous decision-making ability of DRL to enhance the safety of obstacle avoidance, reduce training difficulty, and trial-and-error cost [18].

### 5.3. Establish Standardized Interaction Security Testing Standards

Service, inspection, and other series of robots have been integrated into human living spaces. The evaluation indicators of algorithms should shift from the simple success rate of obstacle avoidance to the social compliance of human–machine integration. To fill the gap of isolated testing scenarios and the lack of a unified yardstick in the academic field, in the future, testing technology standards should be formulated and implemented, which should cover detailed testing benchmarks such as dynamic warning zone response [69] and crowd risk perception quantification [70], providing a security and compliance framework and audit basis for DRL algorithms to move from the laboratory to large-scale commercial deployment.

### 5.4. Lifelong Learning and Cloud-Edge Collaboration for Continuous Adaptive Learning

Complex indoor environments are constantly changing over time. The performance of existing offline training models will decline with environmental drift after deployment. The future DRL navigation obstacle avoidance model should introduce the Lifelong Learning mechanism [82] and the cloud-edge collaborative computing architecture [83]. Specifically, the airborne edge terminal uses a lightweight network to be responsible for high-frequency real-time obstacle avoidance, while the cloud server collects environmental anomaly data for asynchronous retraining. This continuous adaptive ability will be the key to endowing mobile robots with true intelligence.

### 5.5. Bridging the Sim-to-Real Gap for Robust Deployment

Although DRL demonstrates excellent performance in simulated obstacle avoidance, migrating these learned policies to physical platforms remains a critical bottleneck [84]. Due to severe domain shifts, real-world deployment inevitably violates the idealized assumptions of simulators. On a physical chassis, hardware imperfections such as unpredictable sensor noise, limited field of view, action delays, and wheel slip introduce structural instability. Furthermore, standard simulators typically model dynamic pedestrian behaviors mechanically, failing to capture the non-stationarity and social compliance of real human crowds. These perceptual and kinematic misleading factors can cause the robot to exhibit abnormal behaviors, subsequently breaching safety-critical constraints, and ultimately leading to the failure of the obstacle avoidance task [85].

To close the sim-to-real loop and ensure policy reliability, future research must shift from offline pre-training toward an online adaptive paradigm. First, advanced domain randomization must evolve beyond basic geometric randomization to incorporate action delays, wheel friction variations, and non-Gaussian sensor noise distributions within the training cycle, thereby ensuring the network can develop passive robustness against unmodeled real-world disturbances [86]. Second, representation alignment and feature disentanglement are crucial for addressing the challenges of POMDP. By aligning the latent spaces of synthetic and physical sensor inputs, state estimation errors caused by sensor-model mismatches can be systematically filtered, enabling the robot to be more certain of its pose information in the real environment [87]. Finally, reward functions must explicitly parameterize physical delays and hard safety boundaries, coupled with lightweight online policy refinement mechanisms, allowing for real-time tactical fine-tuning upon physical deployment [84].

## 6. Conclusions

This paper first expounds the theoretical basis of the DRL algorithm applied for robot obstacle avoidance in indoor dynamic environments and classifies the classical algorithm systems. Through in-depth analysis of the design ideas of classic algorithms such as DQN, PPO, SAC, and TD3, it summarizes the scene adaptation of different algorithms for robot dynamic obstacle avoidance. It aims to provide a reference for researchers in the field when choosing benchmark algorithms. In addition, the article analyzes the algorithm development of DRL in addressing the core challenges of robot obstacle avoidance in indoor dynamic environments and elaborates in detail on the latest breakthroughs made by the improved algorithm in four aspects: value assessment optimization, spatio-temporal multimodal perception data fusion, hybrid control, and interactive safety perception. The research shows that DRL integrates the powerful high-dimensional feature extraction capability of neural networks with the reinforcement learning decision optimization mechanism, providing an efficient and flexible solution for the design of robot obstacle avoidance systems in complex dynamic environments.

Even though significant achievements have been made in current research, the existing technologies still need to establish more complete theoretical support in handling extremely high-dimensional complex dynamic scenarios, providing reasonable explanations for hierarchical hybrid intelligent control, and ensuring interaction security.

Looking ahead, to ensure the stable and reliable deployment of DRL algorithms in the real physical world, future research should focus on the following core dimensions: breaking the barriers of sensors to achieve a more accurate presentation of the spatio-temporal environment; improving the theory of algorithm security and interpretability; carrying out lifelong learning and adaptive learning for robots to enable them to achieve embodied intelligence. With the continuous exploration and efforts of researchers, significant breakthroughs are bound to be made in the theory of robot decision-making and learning, laying a solid technical foundation for the large-scale application of DRL in the field of intelligent robots.

## Figures and Tables

**Table 1:** Summary of key findings in related survey literature.

References	Key Findings
[20]	This paper categorizes existing dynamic path planning methods into four distinct paradigms: space-based, time-based, environment-based, and learning-based approaches, with a primary focus on outlining the evolutionary trajectory from classical techniques to advanced state-of-the-art methods.
[21]	This article focuses on the utilization of artificial intelligence to assist mobile robot navigation, providing a comparative analysis of the strategic differences among various AI-driven methodologies.
[22]	This paper emphasizes how DRL algorithms enhance robotic exploration capabilities, further elaborating on specific implementations and the potential development of DRL in robotic attitude control tasks.
[23]	Starting from the foundational principles of DRL algorithms, this paper explores their application and integration within mobile robot path planning.
[24]	This study summarizes the performance, functional characteristics, and structural differences in DRL algorithms across multiple typical robotic navigation tasks.

**Table 2:** The differences between MDP and POMDP.

Tuple Elements	MDP	POMDP
Core Tuple	<S,A,T,R,γ>	<S,A,T,R,γ,Ω,O>
State Observability	Fully observable	Partially observable
Applicability	Ideal simulation scenarios only	Real-world physical scenarios

**Table 3:** Summary table of classification and scenario adaptability of basic algorithm systems.

Algorithm Category	References	Base Algorithm	Scenario Applicability
Value-based	[32]	DQN	Suitable for simple indoor obstacle avoidance scenarios with low-dimensional discrete action spaces.
Policy-based	[36]	TRPO	Suitable for scenarios where stability requirements outweigh real-time performance.
Policy-based	[30]	PPO	Suitable for continuous control scenarios requiring a balance between training stability, sample efficiency, and computational cost.
Actor-Critic Architecture	[15]	DDPG	Suitable for continuous control of mobile robot linear/angular velocities; well-adapted to low-dimensional observation spaces.
	[32,39]	A3C/A2C	Suitable for multi-scenario parallel training and multi-scenario generalization training.
	[40]	SAC	Strong exploration capability and robustness; suitable for complex dynamic indoor obstacle avoidance scenarios.
	[42]	TD3	Addresses DDPG’s training instability and overfitting issues in dynamic environments; suitable for complex dynamic indoor obstacle avoidance scenarios.

**Table 4:** Summary of core challenges and solutions.

Core Challenge	Algorithm Subcategory	Algorithms	Solution
Value Evaluation Optimization	Architecture Decoupling	Double DQN [46], Dueling DQN [47]	Dual networks and advantage functions are adopted to solve overestimation bias and low evaluation efficiency under redundant actions.
	Replay and Reward Optimization	DM-DQN [48], PER-D2MQN [49], RND3QN [50]	Prioritized experience replay and multi-step bootstrapping are introduced to solve low sample efficiency under sparse rewards.
	Hybrid Exploration and Curiosity	NM-TD3 [51], ASAC [52], LP-TD3 [53]	Hybrid noise and intrinsic curiosity modules are combined to solve the robot’s fear of exploring unknown regions.
Spatio-temporal Multimodal Perception	Temporal Prediction	GRU-DDPG [54], SAC-LSTM [55], BMTD3 [56]	Temporal prediction modules are introduced to overcome the inability to predict future trajectories of dynamic obstacles in POMDP environments.
	Cross-Modal Feature Fusion	CNN-DQN [57], CMADRL [58]	RGB-D and LiDAR data are fused to extract high-dimensional semantic features, eliminating blind spots inherent in single sensors.
	Attention-Enhanced Prediction	Attention-Enhanced PPO [59], GAP_SAC [60], CAM-RL [61], Spatio-temporal Transformer DRL [62]	Gated/multi-head and global self-attention mechanisms are adopted to accelerate key threat extraction in dense crowds and realize active obstacle avoidance.
Hierarchical Hybrid and Expert Experience Control	Expert Prior Guidance	IDDPG-IAPF [63], VDPF-TD3 [64]	Traditional obstacle avoidance knowledge is embedded into the network as prior information to solve blind collisions in early training and local optima.
	Hybrid Control	A*-DQN-DWA [65], SAC-PID [66], CNN-DQN-B [57]	Traditional algorithms are integrated to resolve kinematic constraint shortages and trajectory oscillations in pure end-to-end control.
	Task Decoupling	SSRL-PPO [67], HRL [68]	It alleviates reward conflicts among multiple tasks and slows generalization to novel environments.
Interactive Safety Perception	Safety Constraint	DRL with Dynamic Warning Zones [69], DRL with Human Risk Predictor [70], Risk-Aware DRL [71], DSRL [72]	Hard risk thresholds are defined based on a constrained MDP to provide a rigorous mathematical safety lower bound for black box decisions.
	Interactive Response Enhancement	Self-Supervised DRL [73], IRL [74], Zero-Shot Hierarchical DRL [75]	Control policies are learned from raw data to improve slow responses to sudden human behaviors.
	Interactive Collaboration Compliance	Voronoi-DRL [76], MADDPG-LSTM Actor [77], MARL [78]	Temporal multi-agent networks and spatio-temporal social encoders are introduced to ensure compliant human–robot and multi-robot collaboration.

**Table 5:** Qualitative summary of algorithms in various obstacle avoidance scenarios.

Algorithm	Environment Type	Success Rate	Convergence Behavior (Episodes)	Comparison Algorithms
Double DQN [46]	Atari 2600 games	The normalized hyperparameter score increased from 233% to 617% on Road Runner	/	Reduces the overestimation of DQN.
DM-DQN [48]	Static and dynamic obstacle environments	67.6%	Approx. 120	Improves the success rate by 18.3% compared with DQN.
PER-D2MQN [49]	Static, dynamic and complex environments	65.8% (static), 52.7% (dynamic), 44.6% (complex)	Approx. 250	Outperforms the comparison algorithms in all obstacle scenarios.
NM-TD3 [51]	Complex static and dynamic environments	92% (static), 82% (dynamic)	Approx. 800	Improves the success rate by 37–28% compared with the original TD3.
SAC-LSTM [55]	Dynamic indoor environments	100% (obstacle-free), 95.5% (static), 89% (dynamic)	Approx. 130 (obstacle-free), Approx. 500 (static), Approx. 600 (dynamic)	Outperforms SAC in all scenarios.
GAP_SAC [60]	Dynamic and narrow environments	96% ((simple), 95% (normal), 93% (complex)	/	Outperforms the comparison algorithms in all obstacle scenarios.
CAM-RL [61]	Cross-shaped pedestrian flow environments	100% (5 people), 99% (10 people),	/	Outperforms CADRL, LSTM-RL and SARL.
VDPF-TD3 [64]	Complex dynamic environments	Effective path planning performance in dynamic environments	Path length shortened by a factor of 0.951	Outperforms the comparison algorithms in all obstacle scenarios.
A*-DQN-DWA [65]	Complex dynamic environments	99.36%	Approx. 100	Outperforms DWA and DQN.
SSRL-PPO [67]	Static and dynamic obstacle environments	Significantly improved obstacle avoidance performance	/	Outperforms hierarchical reinforcement learning.
Risk-Aware DRL [71]	High-density crowd environments	98.0% (10 people), 93.2% (20 people), 90.0% (25 people)	/	Outperforms ORCA, DS-RNN and CrowdNav++.
Zero-Shot Hierarchical DRL [75]	Unseen complex environments	31.88%	Approx. 480	Outperforms traditional HRL (16.28%).
MARL [78]	Dynamic environments with pedestrians	98.8% (5p3r), 99.8% (10p3r), 92.6% (20p3r)	/	Outperforms the comparison algorithms in all scenarios.

## Data Availability

No new data were created or analyzed in this study. Data sharing is not applicable to this article.

## Abbreviations

The following abbreviations are used in this manuscript:

DRL	Deep Reinforcement Learning
AGVs	Automated Guided Vehicles
APF	Artificial Potential Field
DWA	Dynamic Window Approach
MPC	Model Predictive Control
TEB	Timed Elastic Band
VO	Velocity Obstacle
MDP	Markov Decision Process
POMDP	Partially Observable Markov Decision Process
MDPs	Markov Decision Processes
DQN	Deep Q-Network
CNNs	Convolutional Neural Networks
TD errors	Time Difference errors
TRPO	Trust Region Policy Optimization
PPO	Proximal Policy Optimization
D_KL_	Kullback–Leibler Divergence
DDPG	Deep Deterministic Policy Gradient
DPG	Deterministic Policy Gradient
OU noise	Ornstein-Uhlenbeck noise
A3C	Asynchronous Advantage Actor-Critic
A2C	Advantage Actor-Critic
SAC	Soft Actor-Critic
TD3	Twin Delayed Deep Deterministic Policy Gradient
DDQN	Double DQN
PER	Priority Experience Replay
ICM	Intrinsic Curiosity Module
BiGRU	Bidirectional Gated Recurrent Units
SLAM	Simultaneous Localization and Mapping

## Appendix A

Table A1 of the Table presents the algorithm architecture diagram corresponding to the algorithms listed in Table 5 of Section 4 of the review. These visual representations aim to enhance readers’ comprehension of the architectural design and data flow associated with each algorithm.

**Table A1:** Algorithm architecture atlas.

References	Algorithm	Algorithm Architecture
[48]	DM-DQN	
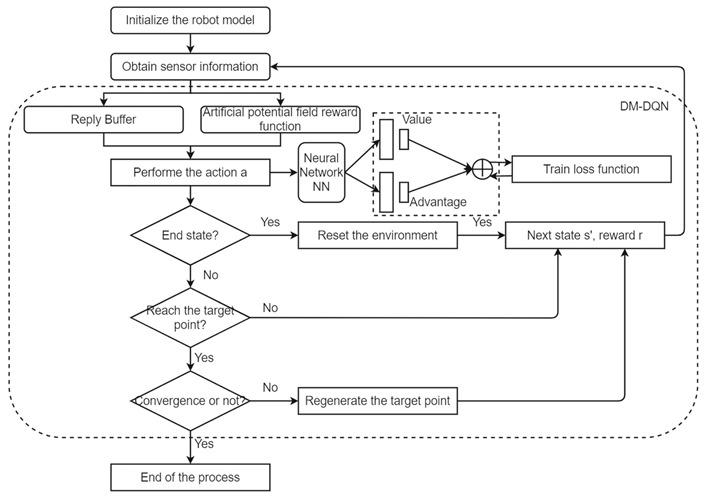
[49]	PER-D2MQN	
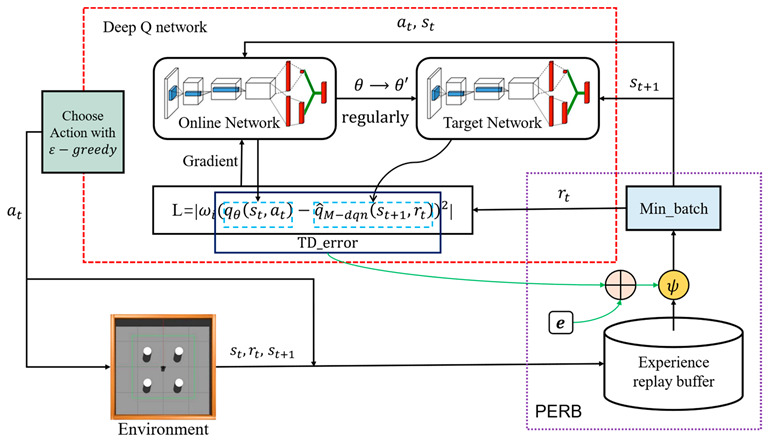
[51]	NM-TD3	
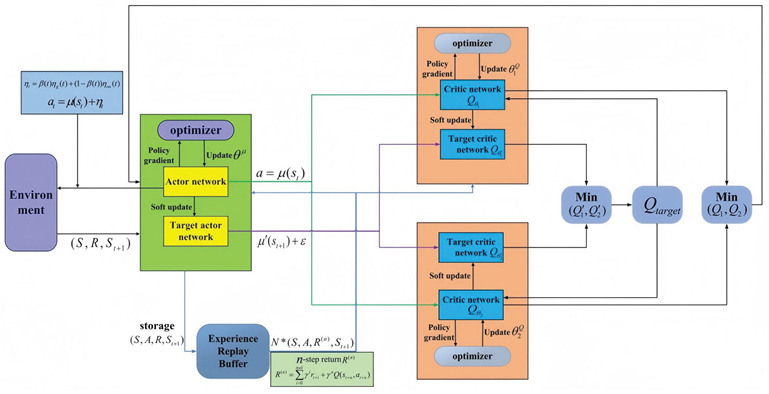
[55]	SAC-LSTM	
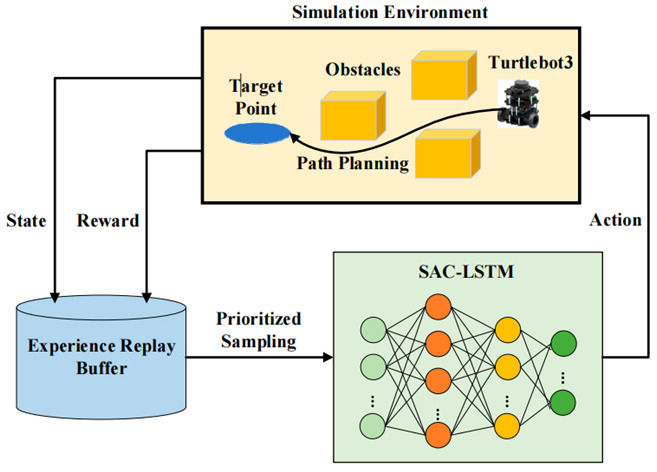
[60]	GAP_SAC	
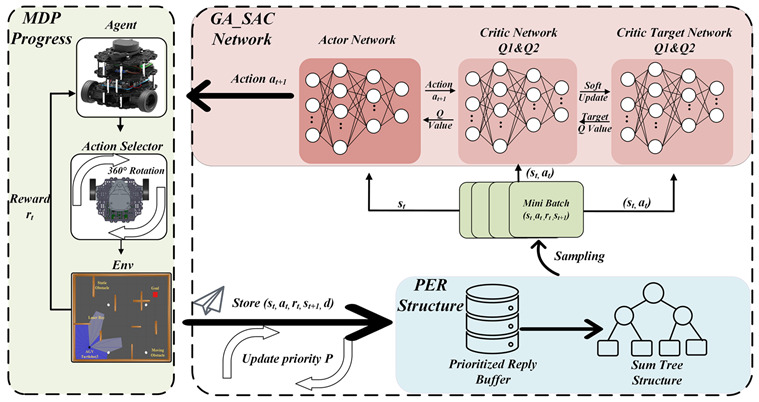
[61]	CAM-RL	
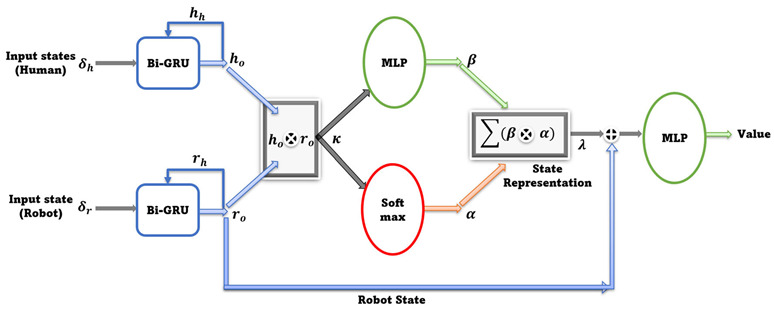
[64]	VDPF-TD3	
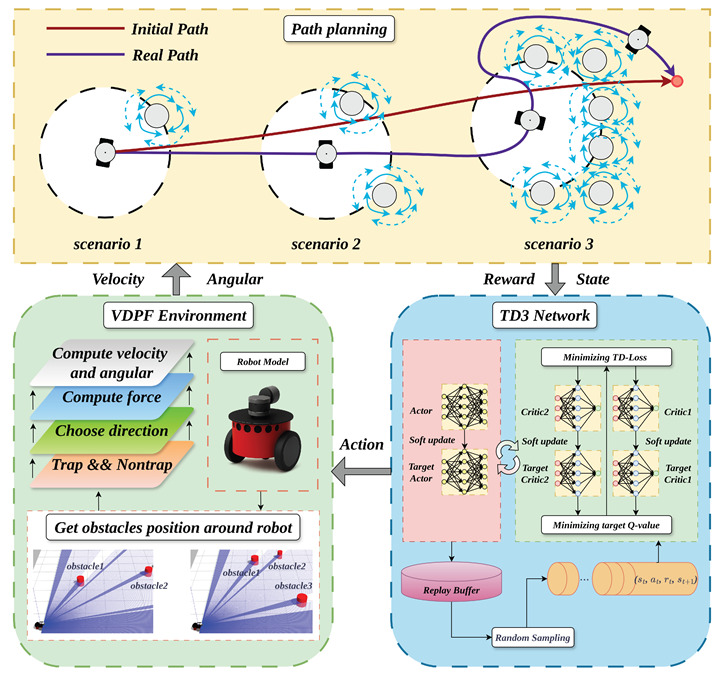
[65]	A*-DQN-DWA	
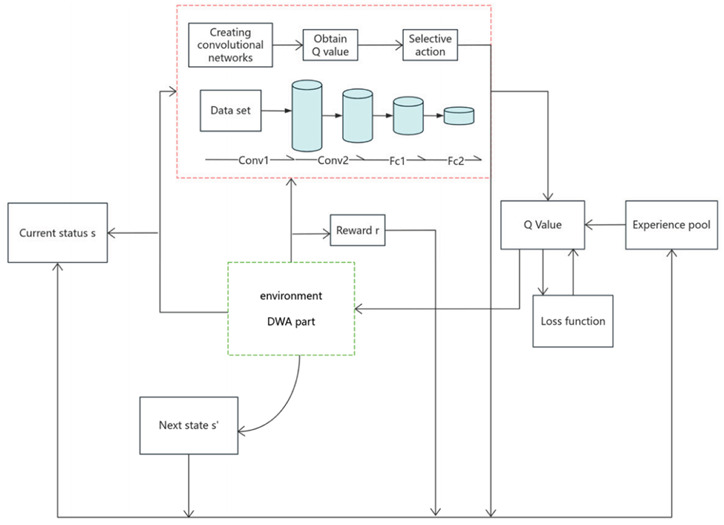
[67]	SSRL-PPO	
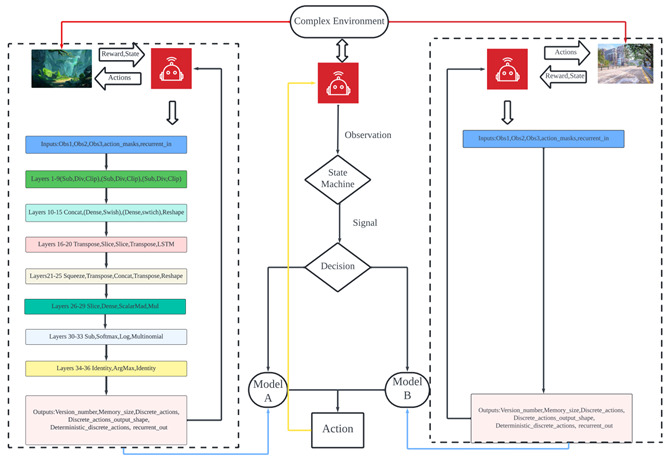
[71]	Risk-Aware DRL	
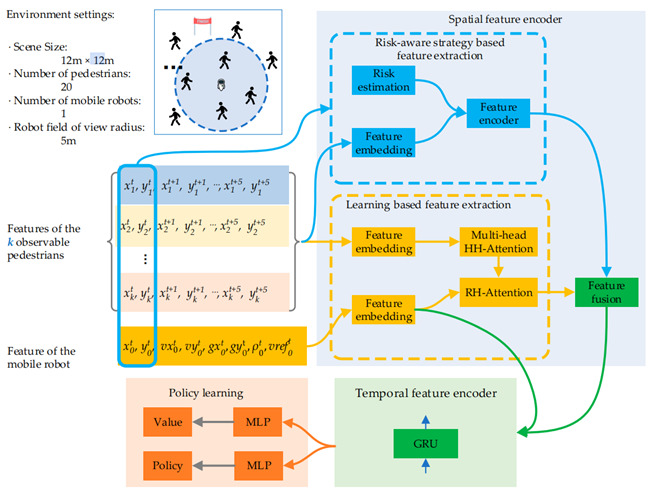
[75]	Zero-Shot Hierarchical DRL	
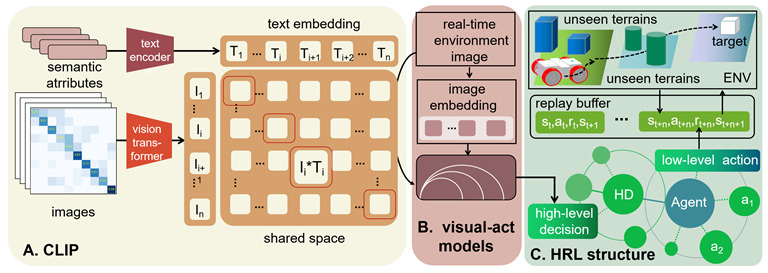
[78]	MARL	
